# Emerging Treatment Strategies for Diabetes Mellitus and Associated Complications: An Update

**DOI:** 10.3390/pharmaceutics13101568

**Published:** 2021-09-27

**Authors:** Vijay Mishra, Pallavi Nayak, Mayank Sharma, Aqel Albutti, Ameen S. S. Alwashmi, Mohammad Abdullah Aljasir, Noorah Alsowayeh, Murtaza M. Tambuwala

**Affiliations:** 1 School of Pharmaceutical Sciences, Lovely Professional University, Phagwara 144411, Punjab, India; pallavinayak97@gmail.com; 2 Faculty of Pharmaceutical Sciences, PCTE Group of Institutes, Ludhiana 142021, Punjab, India; 3 SVKM’s NMIMS School of Pharmacy & Technology Management, Shirpur 425405, Maharashtra, India; mayank2306@gmail.com; 4 Department of Medical Biotechnology, College of Applied Medical Sciences, Qassim University, Buraydah 51452, Saudi Arabia; 5 Department of Medical Laboratories, College of Applied Medical Sciences, Qassim University, Buraydah 51452, Saudi Arabia; Aswshmy@qu.edu.sa (A.S.S.A.); Mjasr@qu.edu.sa (M.A.A.); 6 Biology Department, College of Education, Majmaah University, Majmaah 11932, Saudi Arabia; n.alsowayeh@mu.edu.sa; 7 School of Pharmacy and Pharmaceutical Sciences, Ulster University, Coleraine BT52 1SA, UK; m.tambuwala@ulster.ac.uk

**Keywords:** diabetes mellitus, blood glucose level, insulin, treatment strategies, nanocarrier

## Abstract

The occurrence of diabetes mellitus (DM) is increasing rapidly at an accelerating rate worldwide. The status of diabetes has changed over the last three generations; whereas before it was deemed a minor disease of older people but currently it is now one of the leading causes of morbidity and mortality among middle-aged and young people. High blood glucose-mediated functional loss, insulin sensitivity, and insulin deficiency lead to chronic disorders such as Type 1 and Type 2 DM. Traditional treatments of DM, such as insulin sensitization and insulin secretion cause undesirable side effects, leading to patient incompliance and lack of treatment. Nanotechnology in diabetes studies has encouraged the development of new modalities for measuring glucose and supplying insulin that hold the potential to improve the quality of life of diabetics. Other therapies, such as β-cells regeneration and gene therapy, in addition to insulin and oral hypoglycemic drugs, are currently used to control diabetes. The present review highlights the nanocarrier-based drug delivery systems and emerging treatment strategies of DM.

## 1. Introduction

Diabetes mellitus (DM) is a chronic metabolic disorder and one of the major causes of morbidity globally [1,2]. It is among the top three non-transmissible conditions, responsible for more than 80% of deaths related to non-transmissible illnesses and the top 10 causes of death worldwide. Its global presence has expanded rapidly in recent decades [3].

**Figure 1:**
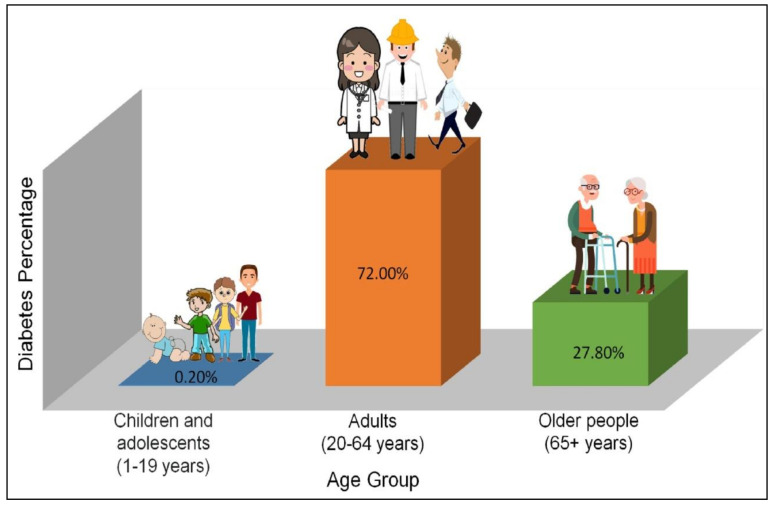
Statistics of diabetic persons in different age groups.

According to 2019 statistics of the International Diabetes Federation (IDF), three out of four people living with diabetes (463 million people) are of working age (i.e., aged between 20 and 64). In 2030 this number will increase to 578 million, and by 2045 to 700 million [4]. An estimated 111 million people aged over 65 have diabetes. In this age group, one in five adults are reported to suffer from diabetes. Approximately 1.1 million children and adolescents (under 20 years old) have type 1 DM (T1DM) (Figure 1). There is also some confirmation that in certain counties, type 2 DM (T2DM) is rising between many children and young adults, but it is not presently possible to determine the statistics due to insufficient data [5]. 

**Figure 2:**
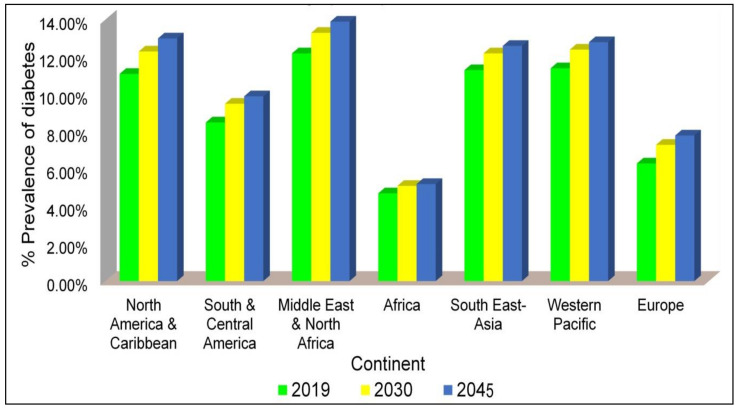
Predicted prevalence of diabetic patients in the year 2019, 2030, and 2045 in different continents.

As per geographical analysis, the IDF Western Pacific region shows the highest age-adjusted prevalence of diabetes: 163 million, 197 million, and 212 million in adults for the year 2019, 2030, and 2045, respectively. On the contrary, the IDF Africa (AFR) region has the lowest age-adjusted rates: 19 million, 29 million, and 47 million for the same estimated years [4], partially due to malnutrition, low levels of obesity, and urbanization. Nevertheless, in this region, by 2045, the number of diabetic people is expected to rise by 143%, the highest increasde in all regions over that time (Figure 2) [5].

**Figure 3:**
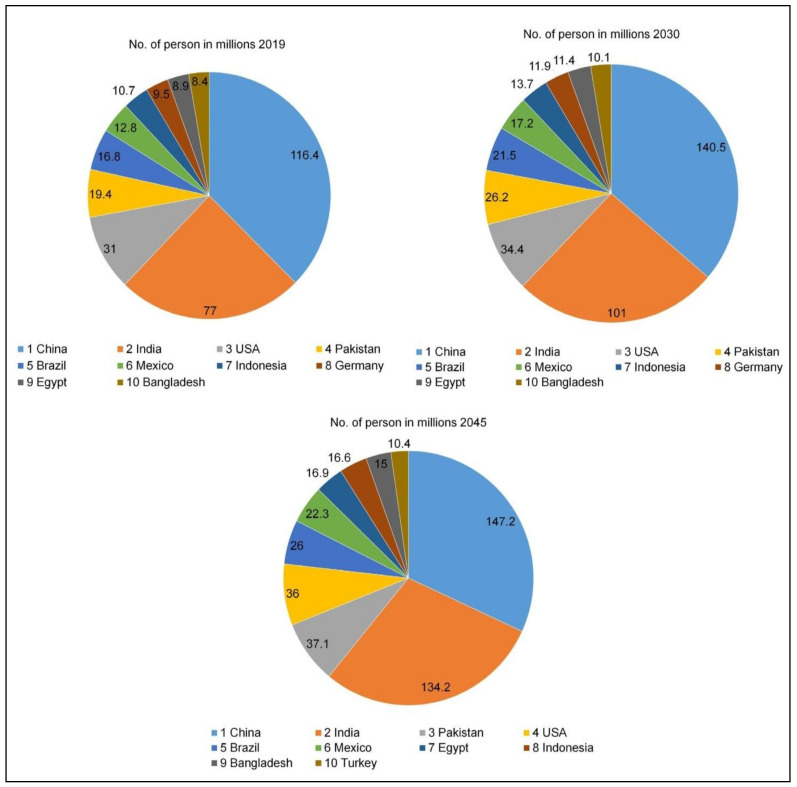
Top 10 countries with the estimated number of diabetic persons in the year 2019, 2030, and 2045.

Throughout 2019, China (116.4%), India (77%), and the United States of America (31%) were the nations with the highest number of adults with diabetes, with figures expected to remain at 140.5%, 101%, and 34.4%, respectively, until 2030. As per the 2045 estimation, China (147.2%) will be in first position, followed by India (134.2%) and Pakistan (37.1%) (Figure 3) [5].

### Diabetes Mellitus: An Insight

The term Diabetes (meaning siphon or to go through), referring to chronic polyuria, one of the most popular features of diabetes, was coined by the Greek-Roman physician, Aretaeus of Cappadocia around the years 30–90 CE. The earliest DM account is derived from the Ebers Papyrus; the assumed Egyptian physician Hesi Ra had published work on the subject of the disease condition around 1550 BCE. The Indian doctors established the earliest clinical sample reported for DM [1].

Diabetes is a chronic disease condition that develops when the pancreas no longer produces insulin or when the body does not utlize the produced insulin properly. Lifestyle consequences and metabolic dysfunctioning cause DM [6]. Three main categories of diabetes exist: T1DM is triggered by an autoimmune reaction where the immune system of the body destroys the insulin-producing β-cells of the pancreas. The most prevalent type of diabetes is T2DM. Hyperglycemia reflecting high blood glucose levels (BGL) is primarily caused by the body’s cells being unable to respond entirely to insulin, a condition called insulin resistance. Gestational diabetes (GDM) contains elevated BGL which can occur during pregnancy (although most likely after week 24) and typically stops after childbirth.

## 2. Oral Antidiabetics: Currently Available Therapy

**Figure 4:**
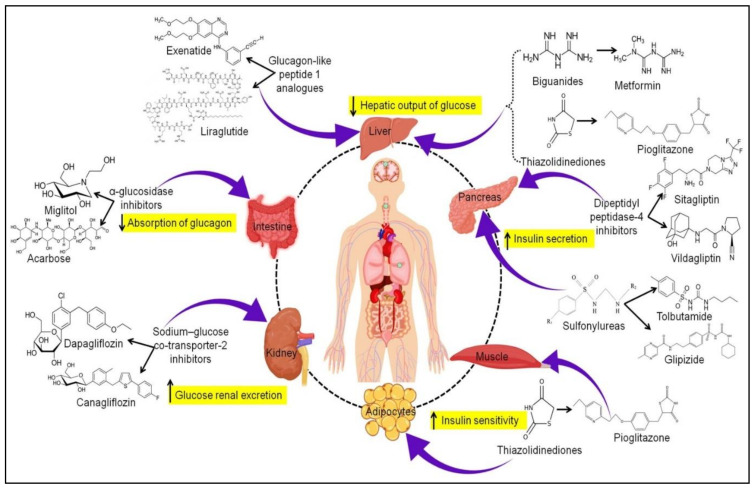
Pictorial representation of different categories of antidiabetic drugs and their mode of action.

Different categories of antidiabetic drugs are currently in practice to manage DM (Figure 4).

### 2.1. Biguanides

Metformin (MET) is recommended as the best option for antidiabetic monotherapy. It increases insulin sensitivity and decreases hepatic glucose production. This drug helps to shape and shows a sensible decrease of triglyceride and serum LDL cholesterol [7]. It activates one of the enzymes associated with expressing genes of hepatic gluconeogene, referred to as adenosine monophosphate-activated protein kinase (AMPK), as well as obstructing enzymes and the mitochondrial complex 1, as well as glycerophosphate dehydrogenase (GPDH) in the mitochondria [8]. This leads to a decrease in the HbA1c and glucose levels. It does not influence β-cells if no reduction in weight is observed. The insulin sensitivity in muscles does not indicate a significant advancement concerning HbA1c levels, which will rise gradually after the initial fall.

Biguanide therapy has been widely used for the T2DM treatment [9]. However, various drawbacks of MET have severely hampered its therapeutic efficacy, including inadequate intestinal absorption, high dose and frequent dosage administration requirements due to its short half-life. To overcome these drawbacks, many nano-DDS have been rationally developed for efficient MET delivery [10].

### 2.2. Sulfonylureas

The sulfonylurea category of antidiabetic drugs activates endogenous insulin secretion from pancreatic β-cells. The typical examples of this category are Tolbutamide (First generation) and Glibenclamide, Glyburide, Glipizide, Gliclazide, and Glimepiride (Second generation). Their main targets are adenosine triphosphate-sensitive potassium channels (KATP) on β-cells and only display potential in residual pancreatic β-cells [11]. Sulfonylurea does not contain well-established defensive consequences for β-cell action and can accelerate β-cells function [12]. Subsequently, there is an initial fall in BGL and an increase in HbA1c concentrations. The BGL concentrations decrease by 20%, while HbA1c levels decrese by 1–2%. The undesirable symptom is weight gain [13].

### 2.3. Thiazolidinediones

Thiazolidinediones (TZDs) are peroxisome proliferator-enacted receptor γ (PPR-γ) activators that perform their action by improving insulin affectability in the liver adipocytes and heart muscles [14]. These are utilized as a treatment protocol for insulin-resistant patients with T2DM [15]. The general side effect of TZD is increased body weight [16]. In T2DM patients, TZD may diminish the width of their carotid arteries. Recently, the FDA has excluded rosiglitazone because of the large number of cardiovascular events; however, the prohibition has since been lifted. The typical examples of this category are Pioglitazone and Rosiglitazone, which are contraindicated in patients with class III and IV heart failure. However, it is tolerated in patients with severe renal impairment [17].

### 2.4. Dipeptidyl Peptidase-4 Inhibitors

Dipeptidyl peptidase-4 (DPP4) inhibitors or gliptins are current treatment agents, which act by restraining the enzyme DPP4. The typical examples in this category are Sitagliptin, Vildagliptin, Saxagliptin, Alogliptin, and Linagliptin. The enzyme inhibition is responsible for neglecting the incretin hormones inactivation: for example, glucagon-like peptide 1 (GLP-1) and gastric inhibitory polypeptide (GIP), associated with controlling glucose homeostasis physiologically [18,19]. The GLP-1 and GIP stimulate insulin synthesis from pancreatic β-cells [20]. Additionally, GLP-1 reduces the glucagon secretion from pancreatic α-cells. Their outcomes result in enhanced glycemic control in patients with T2DM. These drugs show less side effects and lower risk of hypoglycemia [21]. 

### 2.5. Glucagon-like Peptide 1 Analogs

GLP-1 analogs are incretin-based treatments that enhance insulin secretion via a glucose-dependent style, decline the glucagon secretion and reduce the hepatic glucose production [22]. Despite this, these agents are not tolerable as DPP4 inhibitors. Research showed a decrease in HbA1c level and stimulated weight reduction [23]. The endothelial dysfunctions were rectified, such as delayed gastric empty time, better lipid profiles, and reduced blood pressure [24]. There is also some advanced information that expresses the positive impacts of incretin-based treatments on inflammation (by decreasing levels of responsive protein), sleep, the sensory system, and cardiovascular and hepatic health [25]. The typical examples in this category are Exenatide and Liraglutide.

### 2.6. Sodium-Glucose Co-Transporter-2 Inhibitors

Sodium-glucose co-transporter-2 (SGLT2) inhibitors are also called gliflozins, which suppress the sodium uptake resulting in glucose reduction through the kidneys via hindering the uptake of glucose in proximal tubules of the renal nephron [26]. This class includes Dapagliflozin, Empagliflozin, and Canagliflozin. These agents are utilized in patients with diabetes since they act freely on insulin [25]. These drugs can improve the β-cell capacities, upgrade insulin affectability and enhance glucotoxicity because of glucosuria. They can diminish levels of HbA1c by 0.5–1%, and lower weight and BP [27]. The observed side effects are urinary tract diseases, genital mycotic contaminations, especially in females, and the volume consumption linked manifestations [28]. Additional caution should be taken when recommending SGLT2 inhibitors to older patients and those taking diuretics.

## 3. Emerging Treatment Strategies 

### 3.1. Oral Hypoglycemics Incorporated Nanocarrier-Based Treatment

Nanotechnology has excellent applications in the development of a drug delivery system (DDS). The majority of biological performances rely on nanoscale units such as ribosomes and viruses [2]. Nanoparticles (NPs) can directly interact with subcellular entities, which can activate intracellular events. The nanocarriers-based therapeutic DDS is receiving more attention than conventional DDS due to their encouraging applications. As nanocarriers have a greater surface-to-volume ratio, more of the drug surface can come into body contact in the same drug concentration. Therefore, the same dose can make the DDS more successful, and a reduced amount can diminish the toxic effects of drugs. Furthermore, nanocarriers show tunable exterior functionalities for various therapeutic drugs and targeting modalities [3].

Researchers are highly engaged with nanocarriers in the treatment and management of diabetes mellitus due to the limitations of pharmacological therapy and the superiorities of NPs in drug administration and imaging [29]. Liposomes, polymer-based NPs, and inorganic NPs are the most common nano-based drug delivery systems in DM management. Among them, diverse polymer-based NPs including nanocapsules, dendrimers, micelles, and nanospheres, are established as suitable drug carriers. These nanocarriers are potentially advantageous in many aspects, such as improving their stability by overcoming different biological barriers in vivo, increasing bioavailability and protecting drugs from enzymatic degradation. They can also serve as an adaptive automated system to simulate endogenous insulin supply and display a non-linear response to an external signal, lowering the risk of hypoglycemia and increasing patient compliance. Furthermore, they particularly show excellent performance in delivering drugs to specified areas, as well as sustaining and controlling drug release within targeted sites over a prolonged period of time, which could decrease adverse effects while maximising therapeutic impact [30]. Furthermore, due to their unique photoluminescent features, quantum dots (QDs) and metal oxide, NPs are frequently used in the detection of pH and chemical analytes, as well as imaging in drug delivery. Simultaneously, polymer material properties including mean particle size and polydispersity, surface electrical charge, and hydrophilicity of NPs are essential for the delivery of anti-diabetic drugs [31]. Therefore, it is quite necessary and significant to develop appropriate NP-based delivery systems for effective diabetes treatment. NP-based imaging approaches that evaluate subtle changes in β-cell mass can encourage early diagnosis, and nanotechnology-based insulin delivery strategies are being investigated as novel treatments [32].

NPs could be employed to boost paracellular drug absorption. The hydrophobic surface of NPs is beneficial for epithelial endocytosis. On the other hand, cationic NPs interact with the negatively charged mucus layer limiting their absorption [33]. Simultaneously, the secretory goblet and Paneth cells protect and heal the intestine by producing mucins and antimicrobial peptides. Mucus and epithelial barriers must be overcome by NPs designed for oral insulin administration. Although NPs with neutral and hydrophilic surfaces surpass mucus, and their interaction with epithelial cells may be hindered [34,35]. 

Various nanocarriers have been explored for the safe and effective treatment of diabetes. Different reports based on the utilization of nanocarriers in the treatment of diabetes and associated complications have been represented in Table 1. 

#### 3.1.1. Liposomes

Liposomes consist of an aqueous core and lipid bilayer structure. The aqueous core alongside the lipid bilayer forms a liposome capable of loading hydrophobic and hydrophilic drugs in the system. Liposomes enhance drug solubility and avoid their biological as well as chemical degradation in stored conditions [51,52,53]. Joshi et al. loaded both lipophilic drugs (glipizide) and hydrophilic drugs (metformin) in the same liposome (64 ± 6 nm) utilizing a microfluidics-based production method. The co-delivery of the drug significantly enhanced the release rate of both the drugs; glipizide release extended from 3 to 12%, whereas MET extended from 35 to 65% in 1 h. This was associated with the increased permeability of drug throughout liposomes due to glipizide presence inside the highly curved and small lipid bilayers that modified the lipids packaging density. However, the MET presence in the aqueous phase produces the concentration gradient throughout the lipid layer, which leads to the interruption of the lipid membrane. These two features result in a synchronized and improved drug release, which causes synergistic activity [54]. However, liposomes have some disadvantages, such as constrained biological and physical stability ensuing because of conglomeration, fusion sedimentation, and oxidation, as well as phospholipid hydrolysis. Liposomes can also activate defense responses involving the opsonization and initiation of complement process-based pseudoallergy. Further, complications in sterilization and the production of liposomes on a large-scale restrict their commercialization [55,56].

Antigen-specific tolerizing immunotherapy is considered the optimal strategy to control T1DM, a childhood disease involving autoimmunity toward multiple islet antigenic peptides. Bergot et al. argue that Ag-specific tolerizing immunotherapy is regarded as the optimal strategy to control T1DM, a childhood disease involving autoimmunity toward multiple islet antigenic peptides. After BDC2.5mim/calcitriol liposome administration, the adoptive transfer of CD4+ T cells suppressed the development of diabetes in non-obese diabetic (NOD) and combined-immunodeficient mice receiving diabetogenic splenocytes. After BDC2.5mim/calcitriol liposome treatment and the expansion of ChgA-specific peripheral regulatory T cells, IFN-g production and the development of islet-specific glucose-6-phosphatase catalytic subunit-related protein-specific CD8+ T cells were also observed in the pancreatic draining lymph node, demonstrating bystander tolerance at the site of Ag presentation. Thus, liposomes encapsulating the single CD4+ peptide, BDC2.5mim, and calcitriol induce ChgA-specific CD4+ T cells that regulate CD4+ and CD8+ self-antigen specificities and autoimmune diabetes in NOD mice [57].

Villalba et al. developed liposomes rich in phosphatidylserine (PS), a characteristic of apoptotic cells, filled with insulin peptides to imitate apoptotic β-cells’ capacity of apoptotic cell clearance to cause tolerance. Only PS-liposomes encapsulating insulin peptides reduced T1D occurrence in the Non-Obese Diabetic mouse model. Disease avoidance is associated with reducing the incidence of autoimmune islet damage caused by leukocytes. PS-liposomes have no toxicity or secondary complications. Among the autoantigens, insulin peptides are the best candidates to be encapsulated in liposomes, close to an artificial apoptotic cell, for the safe arrest of autoimmunity in T1D [58].

Anti-inflammatory proprieties of curcumin were useful in various diseases, including DM. Bulboacă et al. measured the comparative anti-inflammatory effect of curcumin solution with liposomal curcumin formulations, i.e., LCC1 (1 mg/0.1 kg body weight) and LCC2 (2 mg/0.1 kg bw) in STZ-induced diabetic rats. The improvement of serum levels of tumor necrosis factor-alpha (TNF-α), Interleukin-6 (IL-6), IL-1β, IL-1α, monocyte chemoattractant protein-1 (MCP-1), normal T cell expressed and presumably secreted proteins (RANTES), as biomarkers for systemic inflammation has been recorded). The authors advocated that liposomal curcumin formulation LCC2 (2 mg/0.1 kg bw) showed optimum therapeutic activity as a pretreatment in STZ-induced DM [59].

#### 3.1.2. Niosomes

Niosomes are self-assembled and bilayered nanostructures made of cholesterol and non-ionic surfactants. The bilayered framework consists of a hydrophilic head (directed towards the aqueous solvent) and a hydrophobic tail (oriented far from the solvent). Their unique structure helps to entrap hydrophobic drugs inside the lipid bilayer and hydrophilic drugs inside the aqueous core [60,61]. 

Thier main point of interest is their sustained drug release, which prompts a reduction in the dose frequency and toxicity. In an investigation, metformin-loaded niosomes showed extended hypoglycemic activity for 6–8 h compared to MET solution, reducing the BGL for only 2–4 h. The constant drug release can be accredited to the hydrophobic phospholipid obstacles of niosomes. The interaction of mucosal adhesion amid niosomes with a positive charge (1,2-dioleoyl-3-trimethylammonium-propane chloride salt) and the mucosal layer with a negative charge are accountable for sustained drug release. MET solution demonstrated a maximum decrease in BGL (~25.21%) with 1 h of T_max_, whereas metformin-loaded niosomes exhibited a reduction in BGL (~45.89%) with 4 h of T_max_ [62]. 

Mohsen and co-authors incorporated glimepiride in niosomes consisting of cholesterol and sorbitan monostearate, Span 60, to enhance the therapeutic efficacy of the drug. An in vivo study showed a 7-fold increment in their bioavailability compared to their saline solution. The niosomal preparation formulated has a similar bioavailability as Amaryl^®^ (commercial drug) and is present in the system for a long time. Niosomes have shown a sustained drug release up to 48 h and achieved their T_max_ in 6 h. In contrast to this, Amaryl^®^ and unbound drugs exhibited a sudden decrease in BGL in 2h accompanied by a drastic decline in its plasma concentration. Ten percent of the initial concentration decreased in 24 h [63]. 

The main drawbacks of niosomes are restricted shelf-life and physical instability. Due to its aqueous character, niosomes have a high possibility of hydrolysis, drug leakage, fusion, and aggregation. Moreover, the production cost is relatively high and often requires specialized instruments. Singhal et al. developed and evaluated the gymnemic acid (GA)-loaded niosomes against streptozotocin-nicotinamide (STZ-NA)-induced diabetic nephropathy (DN) in Wistar rats. Animals provided a formulation containing GA-loaded niosomes and had slightly lower antioxidant and lipid amounts. Furthermore, GA-loaded niosomes significantly decreased pro-inflammatory cytokines such as interleukin (IL-6), the tissue necrosis factor, and fibronectin. The study concluded the benefits of GA-loaded niosomes and described the efficacy of the prepared formulation in regulating serum antioxidant, the lipid profile, and diabetic complications in experimentally induced DN in animals by inhibiting advanced glycation end products and oxidative stress [64].

Embelin is a natural agent with a wide range of pharmacological properties, including antidiabetic efficacy. According to Alam et al. embelin, when formulated as niosomes, gives additional advantages in nanoformulations and can be further utilized for therapeutic usage. The STZ-induced diabetic Wistar rats were tested for antidiabetic function. Superoxide dismutase (SOD), catalase (CAT), thiobarbituric acid reactive substances (TBARS), and glutathione were evaluated in an antioxidant assay (GSH). The streamlined formulation demonstrated a strong hypoglycemic impact equal to repaglinide. Furthermore, significant rises in SOD, CAT, and GSH, as well as a decline in lipid peroxidation, were reported, which confirmed the antioxidant efficacy of the formulation. Thus, it is apparent that the embelin-loaded niosome formulation successfully treated diabetes in Wistar rats [65].

Samed et al. reported a niosomal formulation for the simultaneous encapsulation and release of hydrophobic and hydrophilic antidiabetic drugs. The encapsulation efficiencies for glipizide and metformin hydrochloride were found to be 67.64% and 58.72%, respectively. The drug release experiments performed in buffers at different pH values (mimicking the blood plasma pH, cellular endosomal, and gastric environments) revealed that the drug release followed a linear profile up to 8–10 h, slowed down, and lasted for 12–14 h. Thus, this formulation offers a promising DDS for combinatorial sustained release of antidiabetic drugs [66].

#### 3.1.3. Micelles

Micelles are defined as aggregates of amphiphilic molecules, which can help to solubilize hydrophobic drugs. Moreover, clusters can be formed when the achieved concentration corresponds to the critical micelle concentration (CMC). Their assembling nature and molecular frame are well-defined, and the simple manufacturing process of drug solution is stabilized using micelles [67,68].

Kassem et al. fabricated repaglinide-phospholipid-complex-enriched micelles (RG-PLC-Ms) via the solvent evaporation method. The observations showed that following 7 days of oral route treatment, the micelle preparation decreased the BGL in diabetic-induced rats by 83.02% (from 558.40 to 94.80 mg/dL) compared to a 55.40% decrease (from 543 to 242.20 mg/dL) as a result of the commercial product [69]. 

Additionally, RG-PLC-Ms enhanced insulin levels and serum malondialdehyde and demonstrated an improved lipid profile [70]. The presence of phospholipid in formulation decreased the surface tension amid fabricated compound, and gastrointestinal tract (GIT) fluid led to extended drug permeation and transfer throughout the cell membrane [69,71]. Conversely, the least in vivo stability and constrained drug loading capacity restricted the micelle’s application [72].

Insulin delivery mechanisms for diabetes care have been broadly implemented during the last several decades. Liu et al. examined polymeric micelles with dual responsiveness to glucose and hydrogen peroxide (H_2_O_2_) for insulin transmission. Poly(ethylene glycol)-block-poly(amino phenylboronic ester) (PEG-b-PAPBE) self-assembled the polymeric micelles, with the hydrophilic PEG providing the shell and the hydrophobic PAPBE endowing the polymeric micelles with dual sensibility to glucose and H_2_O_2_. The built-in phenylboronic ester (PBE) could be hydrolyzed by H_2_O_2_ and broken by glucose, which resulted in glucose-responsive insulin release due to polymeric micelles disintegration. The insulin release was further boosted by the co-encapsulation of glucose oxidase (GOx) in the micelles. The Gox mediated catalytic oxidation of glucose generated the H_2_O_2_, which hydrolyzed the PBE. The subcutaneous injection of insulin/GOx-coloaded polymeric micelles to diabetic mice showed a superior hypoglycemic impact in vivo as compared to free insulin or micelles that bore insulin alone. This polymeric micelle with dual glucose and H_2_O_2_ tolerance presented a promising path to diabetes care [73].

Zhu et al. synthesized poly(ethylene glycol)-b-poly(3-acrylamidophenylboronic acid-co-styrene) (PEG-b-P(PBA-co-St) to develop insulin-loaded micelles. In vivo wound healing study was performed on an STZ-induced rat model to assess the wound healing capacity of as-prepared composite hydrogels. Furthermore, the as-prepared composite hydrogels with ILM and EGF demonstrated excellent wound healing ability in fibroblast proliferation, tissue internal structure integrity, and collagen and myofibril deposition. The findings indicated that as-prepared composite hydrogels with ILM and EGF might be a successful candidate for wound healing applications [74].

Oral administration of insulin is the most suitable and attractive as compared to the subcutaneous route but, unfortunately, cannot be utilized for the administration of peptides and proteins due to poor epithelial permeability and enzymatic degradation within the gastrointestinal tract [75]. The oral delivery of insulin presents several challenges, specifically the poor bioavailability resulting from the low intestinal permeability, lack of efficacy, enzymatic degradation, potential toxicity, lack of specificity, side effects. The use of several types of nanomaterials, such as micelles, liposomes, and hydrogels has enabled oral insulin administration to overcome GIT obstacles [76].

Bahman et al. produced a poly-(styrene-co-maleic acid) (SMA) micellar device for oral insulin distribution to address the accelerated degradation of insulin in the stomach, the enhanced absorption in the intestine, and include a physiologically relevant form of insulin to access portal circulation. Animal tests found that orally administered SMA-insulin would cause a hypoglycemic effect for up to 3 h after a single dose. Overall, the findings suggested that SMA micelles were found to be capable of the oral transmission of bioactive compounds such as insulin and might be valuable tools in treating diabetes [77].

#### 3.1.4. Nanoemulsion

Nanoemulsion (NE), in contrast to microemulsion, is a thermodynamically unstable nanosized emulsion [78]. Xu et al. designed Berberine (BBR)-loaded NE to explore the hypoglycemic potency of BBR in STZ-induced diabetic mice. The BGL in diabetic mice was found to be 3-fold less than in BBR-loaded NE treated group than that of BBR alone. This innovative NE offered a strong delivery carrier to boost the hypoglycemic potency of BBR for diabetes therapy [79].

The potential of NE for the oral administration of peptides is still in its early stage. Santalices et al. developed and fully characterized NE intended for the oral administration of hydrophobically modified insulin (HM-insulin). A developed hybrid system of NE co-existed with micelles exhibited 100% HM-insulin association efficiency. The nanosystem showed good stability and miscibility in different bio-relevant media and displayed an acceptable mucodiffusive behavior in porcine mucus. In addition, it exhibited a high interaction with cell monocultures (Caco -2 and C2BBe1 human colon carcinoma Caco-2 clone cells) and cocultures (C2BBe1 human colon carcinoma Caco-2 clone/HT29-MTX cells). Overall, this information underlined the crucial steps to address the intestinal barriers associated with the oral delivery of peptide-based nanoformulations [80].

Okra (*Abelmoschus esculentus*) has potential antidiabetic activity. Djamil et al. created a nanoemulsion of okra extract (NOE) and examined its activity on alloxan-induced DM in mice. For determining the antihyperglycaemic effect of okra extract, 35 male mice (*Mus musculus* L.) were divided into seven groups: a non-diabetic normal control group and six diabetic mice groups (untreated negative control, glibenclamide-treated positive control, and four treatments with okra ethanol extract (OEE) at 200 and 400 mg/kg bw and NOE at 200 and 400 mg/kg bw). The NOE reduced BGL in alloxan-induced hyperglycaemic mice more successfully than OEE did. The NE could improve the antidiabetic activity of okra extract by increasing the penetration of active compounds into interstitial space so that their delivery and bioavailability become higher [81].

In diabetic patients, wound healing remains a challenging clinical problem due to the insufficient inflammatory response and impaired ability to fight infection. Gundogdu et al. evaluated the effects of boronophenylalanine (BFA) and/or Zn-containing NE formulations on wound healing in diabetic rats. Histopathological results demonstrated nearly complete wound healing in BFA and/or Zn-NE treated animals as compared to the untreated diabetic rats. Animals treated with the NE containing a low concentration of BFA demonstrated complete epithelialization and an entirely closed wound area within 14 days. This study showed encouraging results in the wound healing of diabetic rats [82].

#### 3.1.5. Polymeric Nanoparticles

Nanoparticles (NPs) are colloidal DDS of nanoscopic particle size (10–1000 nm in diameter). Polymeric NP present in reservoir systems where the drug is enclosed in a cavity and confined through a polymer film is called a nanocapsule or matrix system, whereas when the drug is dispersed across all the particles it is called nanosphere [83,84]. The primary standpoint of enclosing drugs within nanoarchitectured DDS is their improved bioavailability and circumvented first-pass metabolism. This leads to a decreased dose as well as reduced toxic effects of the drug on non-targeted cells. Targeted or smart DDS utilizing NP diminishes toxic effects, extends the drug concentration in the localized region, and encourages the rapid onset of action [84].

The NPs consisting of poly(lactic acid) (PLA), a biodegradable and biocompatible polymer, are commonly used for drug delivery via the oral route in the treatment of diabetes complications [85]. Despite the various points of interest, NPs also have some toxicity concerns. If NPs are inhaled unintentionally, they can accumulate in different body organs, especially the lungs. For instance, NPs deposited in the respiratory system can cause inflammatory reactions associated with oxidative stress. They can also attain access to the central nervous system (CNS) by inhalation via direct uptake or olfactory receptors across the blood–brain barrier (BBB) [86]. Besides the toxic effects, NPs have some other constraints, such as low shelf-life, unpredictable stability, and high production expenses [87].

Lari et al. synthesized novel cross-linked carboxymethyl chitosan nanoparticles (CMCS NPs) containing MET using the microfluidics (MF) technique and evaluated their performance in diabetes therapy. Researchers demonstrated high encapsulation efficiency (90%) and managed a drug release via CaCl_2_-based crosslinking. Finally, in vivo studies showed that MF MET-loaded CMCS NPs raised body weight by 7.94% and reduced BGL by 43.58% as compared to the free drug in diabetic rats. Furthermore, histopathological results showed that the scale of the pancreatic islets was 2.32 m^2^ and the cell intensity was 64 cells per islet in diabetic rats treated with the MF-based sample. These data were close to those obtained for the healthy rats [88].

Hadiya et al. developed insulin-loaded NPs comprised of various polymers at different compositions and evaluated their BGL lowering ability in diabetic rats following subcutaneous and oral administration. The NPs were well-tolerated after oral administration in rats, as evidenced by measuring the level of alanine aminotransferase, aspartate aminotransferases, albumin, creatinine, and urea. This study indicated that the features and delivery efficiency of nanomaterials could be controlled by utilizing several natural/synthetic polymers and by fine-tuning the combination ratios between polymers [89].

Ribeiro et al. developed insulin-containing chitosan nanoparticles (CNPs) and evaluated their therapeutic efficacy during wound healing in diabetic rats. The study hypothesized that the combination of insulin inside CNPs could activate the wound healing signaling pathway. The productivity of the insulin alliance was 97.19%. These NPs and free insulin (FI) were mixed in a hydrogel (Sepigel^®^) for topical application in the wounds of 72 diabetic rats classified into four groups: Sepigel^®^ (S, control), FI, empty CNPs (ECNPs), and CNPs containing insulin (ICNPs). The animals in each category were grouped into three subgroups (*n* = 6) to determine their clinical indications on days 3, 7, and 14 after therapy started. In the free or ICNPs types, severe fibroplasia was observed. On the 7th day, a significant number of blood vessels were found in the latter. The findings showed that both ECNPs and ICNPs could induce inflammatory cell proliferation and angiogenesis, accompanied by wound maturation [90].

#### 3.1.6. Solid Lipid Nanoparticles

Solid lipid nanoparticles (SLNs) composed of solid lipids (high melting fat matrix) are nanosize (50–1000 nm) colloidal carriers with the capability to improve the bioavailability and solubility of drugs [91].

Anchan et al. formulated chitosan-coated, insulin-loaded SLN for oral administration and investigated their potential as effective alternatives to the subcutaneous injection. At the end of an 8 h trial, the oral administration of chitosan-coated insulin SLN to STZ-induced diabetic rats resulted in a substantial hypoglycemic impact (*p* < 0.05) as compared to groups receiving uncoated insulin-loaded SLN or the oral insulin solution, which was equivalent to subcutaneous insulin. Chitosan-coated SLN can be an efficient oral insulin formulation [92].

Oral insulin administration has been hampered so far due to gastrointestinal enzyme depletion and slow intestinal absorption. Muntoni et al. developed solid oral dosage forms from glargine insulin-loaded nanostructured lipid carriers (NLCs). In STZ-induced diabetic rodents (six animals in each group), the liquid and solid oral formulations were checked for glargine insulin absorption and glucose responsiveness. The liquid and solid formulations had significant hypoglycemic effects in balanced rats, but only capsules were successful in diabetic rats, most likely attributable to gut alterations in these animals. Glargine insulinemia was found to be associated with a glycemic profile. The formulations under investigation demonstrated their potential as oral glucose-lowering agents, particularly when used as capsules [93].

Oroojan et al. studied the impact of myricitrin and myricitrin-containing SLNs on the reproductive system of type 2 diabetic male mice. In diabetic mice, the total antioxidant potential and SOD levels decreased, while myricitrin (10 mg/kg) or all doses of SLN containing myricitrin (1, 3, and 10 mg/kg) increased antioxidant potential and SOD levels (*p* < 0.05). The weight and volume of the testes in the diabetic group were found to be decreased. Treatment with a high dose of myricitrin or all three doses of SLN containing myricitrin restored the luteinizing hormone, follicle-stimulating hormone, testosterone, and sperm count in the diabetic population (*p* < 0.05). Diabetes caused vacuoles and apoptosis in testicular cells, but myricitrin and SLN with myricitrin strengthened them (*p* < 0.05). Diabetes caused reproductive problems by increasing oxidative stress and decreasing antioxidant capacity, whereas the administration of myricitrin or SLN containing myricitrin supported these symptoms. Furthermore, SLN containing myricitrin was more successful than myricitrin alone [94].

#### 3.1.7. Dendrimers

Dendrimers are homogenous well-defined 3-dimensional (3D) structures with tree-like branches. Dendrimers have received extraordinary attention to achieve controlled drug delivery. Dendrimers represent more diversity of chemical structure than poly(etherhydroxylamine) (PEHAM), poly(propylene imine) (PPI), poly(amidoamine) (PAMAM), poly(L-lysine) (PLL) and polyester dendrimers. A tunable toxicity profile and dendrimer targeting are possible with the help of surface groups, so it is expected that, in the near future, researchers will achieve success in developing dendrimer-based novel antidiabetic therapeutics to control the high BGL effectively, as well as the complications associated with diabetes [95].

The delayed wound healing of diabetics contributes to a number of life-threatening problems correlated with the overexpression of matrix metalloproteinases (MMPs). Zhang et al. generated the MMP-2-responsive nanocarriers, HA-pep-PAMAM, by combining PAMAM dendrimer with the polysaccharide hyaluronic acid (HA) through the substrate polypeptide (Gly-PLGLAG-Cys) of MMP-2. The H_2_O_2_ has a dose-dependent effect on the proliferation of BJ and HaCaT cells, with the HA-pep-PAMAM-ASI therapy providing the highest antioxidant potential with MMP-2 pretreatment. To achieve antioxidant benefits, HA-pep-PAMAM-ASI greatly increased GSH levels, thus decreasing reactive oxygen species (ROS). Cell proliferation and migration abilities were enhanced in the MMP-2-pretreated HA-pep-PAMAM-ASI community. When compared to the ASI population, the expression of all wound-repair-related genes was substantially increased in the HA-pep-PAMAM-ASI group, which had an important in vivo therapeutic benefit. As a consequence of the findings, enzyme-responsive MMP-2-loaded PAMAM dendrimers may facilitate wound healing in diabetes and may be a promising biomaterial for diabetic care [96].

Akhtar et al. examined whether the persistent administration of nano-sized PAMAM dendrimers could improve diabetes-induced vascular dysfunction by inhibiting the epidermal growth factor receptor (EGFR)-ERK1/2-Rho kinase (ROCK) pathway, responsible for diabetic vascular complications. Data showed that plain PAMAM dendrimers could modulate EGFR cell signaling cascades in vivo with associated pharmacological effects depending on their physicochemical properties. Thus, PAMAM dendrimers, either alone or in conjunction with vasculoprotective agents, can play a beneficial role in treating diabetes-related vascular complications [97].

For diabetic patients who risk limb amputation, creating a practical approach to enhance the wound healing process is essential. Several growth factors have been suggested as treatment; however, further study is needed to preserve their curative role. Kwon et al. identified a nonviral gene therapy approach for promoting wound healing. Actively proliferating cells of wound tissue were successfully transfected, resulting in a strong VEGF expression. Histological staining indicated that skin wounds in diabetic mice were healed and showed a well-ordered dermal structure within 6 days of injection. This quick and efficient gene therapy approach may be a valuable strategy for treating diabetic foot ulcers [98].

Labieniec-Watala et al. administered the PAMAM G4 dendrimers to diabetic animals from three different routes (intraperitoneally, intragastrically, or subcutaneously), and their hypoglycemic impact was assessed. Intraperitoneal administration has a stronger blood glucose scavenging impacts, although intraperitoneal and subcutaneous routes were the most successful in suppressing the long-term symptoms of hyperglycemia. However, the intraperitoneal injection was correlated with lower survival rates due to carrier toxicity [99].

#### 3.1.8. Carbon Nanotubes

Carbon nanotubes (CNTs) have emerged as promising DDS due to their fascinating physicochemical features. The CNTs can be functionalized with various therapeutically active molecules to explore their biomedical potential. Zaman et al. reported the antidiabetic activity of functionalized CNTs (*f*-CNTs). The multi-walled carbon nanotubes (MWCNTs) were functionalized using 95% concentrated nitric acid (HNO_3_) by refluxing. The functional groups on the surface of MWCNT were investigated by Fourier transforms infrared spectroscopy (FTIR) and X-ray photoelectron spectroscopy (XPS). The high-resolution transmission electron microscopy (HRTEM) was used to directly observe the changes in the surface morphology of MWCNT after its functionalization. The *f*-CNTs showed 41.2% in vitro aldose reductase inhibition activity. Furthermore, the docking study revealed the significant role of the carboxylic group in inhibition activity. This study indicates the potential of MWCNTs in antidiabetic activity [100].

#### 3.1.9. Contribution of Authors to the Nano-Based Diabetes Therapy

The authors of the present review have made significant contributions in nanotechnological advances in the diagnosis and treatment of diabetes to date. Our recently published work presented the crucial scientific progress in copper nanostructure-based fourth-generation glucose sensors (FGGS) over the past 10 years and highlighted the importance of copper nanostructures as advanced electrode materials to develop reliable real-time FGGS [101]. Furthermore, in another recent report, we highlighted the vital roles of plant-based medicines backed by different scientific evidence including their nanocarriers mediated delivery for improved pharmacokinetic and therapeutic actions in the management of diabetes [3].

Our research examining the effect of zinc oxide NPs (ZnONPs) on diabetic nephropathy indicated that ZnONPs could ameliorate the renal damage induced in a diabetic rat model of nephropathy via improving renal functionality; inhibiting renal fibrosis, oxidative stress, inflammation and abnormal angiogenesis; and delaying the development of podocyte injury [102].

It is known that gold NPs (AuNPs) attenuate hyperglycemia in diabetic animal models without any observed side effects. Our research showed that AuNPs treatment prevented diabetes-associated increases in the BGL via the downregulation effect of AuNPs in renal mRNA or the protein expression of transforming growth factor β1 (TGF-β1), collagen IV, fibronectin, vascular endothelial growth factor-A (VEGF-A), and TNF-α [103]. In another study, the anti-hyperglycemic activity of AuNPs in STZ-induced diabetic animal models has been estimated. It is observed that AuNPs cause a reduction in hyperglycemia, decreasing the protein tissue content of TGF-β1 and myocardial mRNA in diabetic cardiomyopathy animal model [42].

Another study from our group showed that insulin-loaded poly(lactide-co-glycolide) (PLGA) NP suspended in structured poly (vinyl alcohol)-borate hydrogel improved the wound healing with better wound injury percentage indices [104]. A research showed that insulin-loaded NP prepared from 10% PEG-PLGA possessed therapeutically useful encapsulation and release kinetics when delivered by the intraperitoneal (i.p.) route [105]. Recently we reported that Glimepiride containing nanosuspension, prepared by antisolvent precipitation, and followed by the sonication method showed a better drug solubility and dissolution profile than nanosuspension prepared by the nanoprecipitaion method [106]. The potential of dendrimers in the delivery of antidiabetic drugs has been reviewed for the effective management of diabetes [95].

### 3.2. Insulin Pump

The National Institute for Health and Clinical Excellence (NICE) Technology Appraisal Guideline TA 151 defined who may gain profit through continuous subcutaneous insulin infusion (CSII) treatment. For children < 12 years and especially for <5 years, CSII treatment is prescribed as the superior therapy alternative [107]. Different new methodologies for insulin delivery have offered excellent advantages over conventional strategies. These methodologies involve insulin delivery via palmary, oral, buccal, nasal, rectal, and ocular routes. The advanced ultra-long insulin analog, the Degludec, is also considered efficient [108].

Insulin pump therapy seeks to mimic physiologic insulin secretion by continuously delivering small quantities of basal rate insulin augmented by prandial boluses. The ability to adjust basal insulin and boluses provides far more precise insulin delivery based on daily variations in individual insulin sensitivity and needs. Insulin pumps are becoming smaller and easier to use. With the rapid advancement of technology, the current accessible pumps are characterised as “smart,” with many additional functions facilitating diabetes care. The ability to modify numerous daily basal and temporary rates addresses transient differences in insulin sensitivity, such as those observed during physical activity or associated illness. Insulin requirement patterns vary throughout the day dependent on age and pubertal stage. Insulin pump therapy allows nearer physiologic basal rate delivery adapted to the individual circadian needs [109].

Insulin pumps enable more precise insulin dosing, greater flexibility in daily self-care, and fewer injections when compared to multiple daily injections (MDI) or Multiple dose injection therapy. Many investigations have found that insulin pumps provide better glycemic control with a 50% reduction in the risk of cardiovascular mortality than MDI [110,111].

Insulin pumps deliver fast-acting insulin continuously (small doses) using a catheter fixed inside the skin. These pumps are smaller in size (like cellphones) attached to socks, pockets, belts, or clothes. Several pumps have electrical memories, various basal metabolic rates, different bolus choices, adjustable menus, alerts, and electrical controls. These surface insulin pumps are comprised of a reservoir (insulin), a small cordless pump, and a computer chip that manages the insulin delivery. This is associated with a small and elastic-plastic tube, ended with a needle embedded simply underneath the skin, closer to the stomach. This implantation set is altered every couple of days. Patients locate the pump to yield a consistent basal or trickle insulin amount persistently for the whole day. The pump releases a bolus insulin dose, which is automated to deliver a lesser quantity of insulin for the entire day. Examples of the insulin pumps model are Animas IR 1000, Cozmo, MiniMed 508, Disetronic, Amigo, MiniMed 512, and DANA Diabecare II [112].

The insulin preparation used in pumps is intended to withstand aggregation and temperature. Miscible, fast-acting insulin analogs (for example, Aspart or Lispro) are suitable for constant infusion. When placed in an external MiniMed pump for seven days at 25 °C, the insulin endured with no precipitation [113]. This therapy needs patients to fill up a reservoir system in the pump each couple of days. Medtronic collaborated with Novo Nordisk to form long-enduring prefilled cartridges of insulin consisting of Novo Log.

According to Diabetes Control and Complications Trial (DCCT), the control of intense glucose could be accomplished by the CSII, insulin pump or an insulin injection. In the US, CSII utilization increased from 15,000 patients in 1993 to 162,000 patients in 2001 [114]. In contrast to insulin injections, CSII yields reduced the variation ability in insulin absorption, and improved bioavailability, distribution, metabolism, excretion of insulin, enhanced HbA1C levels, and diminished hypoglycemia risk. It permitted patients more lifestyle adaptability. The fast-acting insulin absorption utilized in CSII differed by <3% daily in contrast to 19–55% for injected insulin [115].

The CSII pharmacokinetics are superior, as meal sugars are secured by insulin boluses activated by the individual, whereas a continuous basal insulin delivery maintains normal BGL on different occasions. Both dawn phenomena (increase in BGL before waking) and nocturnal hypoglycemia are enhanced by CSII treatment [114]. The meta analysis of 52 research reports including 1547 patients, indicated that the enhanced CSII exerted more control over BGL compared with insulin injections and diminished the frequency of hypoglycemia [116].

In another investigation, the standard HbA1C limit reduced from 8.3 to 7.5% in 3 years in 413 young T1DM patients [108]. Type-II patients who failed to control BGL using an injection can take advantage of CSII therapy. In the single multi-focus experiment of 126 T2DM patients, the CSII diminished the plasma BGL, and the HbA1C value was found to be greater than the insulin injections [117]. Additionally, 97% of patients favored insulin pumps over injections due to precise delivery, better glycemic control, and general well-being [118].

Moreover, implantable pumps were developed that could deliver insulin through the intraperitoneal route [119,120]. This pump delivered a small insulin amount daily. Patients could control the dose by a handheld unit leading to the insulin pump therapy to yield a predefined quantity of insulin. This pump was refilled every 2–3 months. For instance, the Mini-Med Implantable Pump consisting of an improved side port catheter, which used the formulation of insulin with an enhanced stability against agitation and temperature, was also utilized in the European clinics [121].

Various studies on insulin-dependent diabetes patients have shown that insulin transported by an embedded pump is fast and typically absorbed with the vast majority to the entrance system, leading to the delivery of hepatic insulin. Research has shown that excellent glucose control can be accomplished with lesser glycemic fluctuations and fewer hypoglycemia episodes [122,123].

Different studies were performed to estimate the general use of the insulin pump in recent years [124,125]. The survey data of the electronic medical report (EMR) from 2011–2016 showed a rise in the overall use of a pump (61.8%) in contrast to previous records and population-based information as 22% in the mid-2000s was found to be increased to 56.3% by 2012 [126] and are equivalent to the information gathered from multi-site records office in 2013—1488 [127]. O’Connor et al. identified a new factor related to a lower utilization rate of the insulin pump that was not identified previously (families/patients with preferred non-English speaking). Their outcomes also established additional factors earlier related to lesser insulin pump therapy rates. The usage of the insulin pump was related to improved glycemic control at 8.5%. The inclusive pump used has elevated overall groups, but the differences amid socio-demographic groups remains [128].

### 3.3. Pancreatic Islet Cell Transplantation

Pancreatic islet transplantation is a standout amongst the most encouraging therapeutic methodologies for the individual with serious T1DM. The transplantation of designed islet cell sheets has an incredible ability for managing T1DM; it empowers the production of constant neo-islet tissues. Yet, a substantial gathering of islet cell sheets is needed for subcutaneous injection, which aims to inverse the hyperglycemia in the diabetic-induced mice. Saito et al. reported a strategy for producing neo-islet tissues within the diabetic mice’s subcutaneous space via transplanting islet cell sheets fabricated from isolated islet cells [129]. A large islet cell mass (total of 1.1 × 10^7^ cells per animal) was requisite to reach euglycemia in the mice among STZ-induced DM. The insufficiency was caused as the transplanted islet cells were neither functional nor viable due to the inadequate nutrition and O_2_ supply caused by the absence of untimely neovascularization [130].

**Figure 5:**
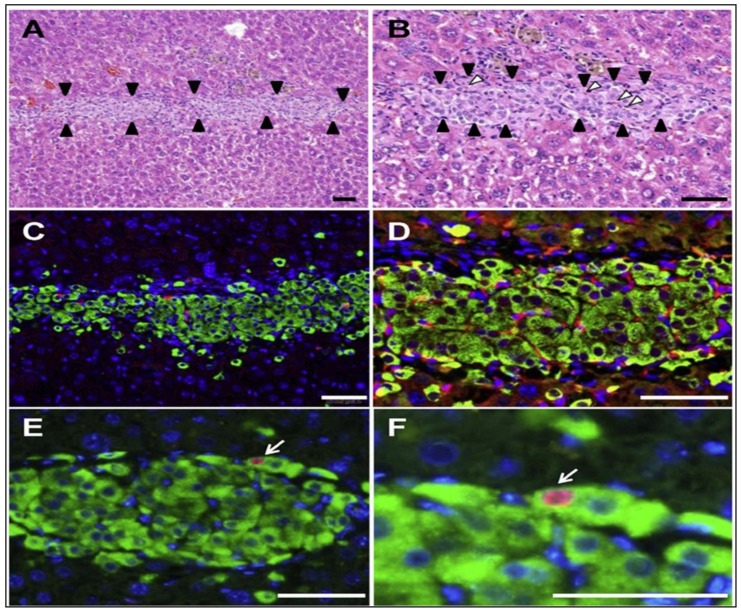
Histological analysis of the islet cell sheets that were engrafted on the surface of the left lateral lobe of the liver and covered by the median lobe of the liver. (**A**,**B**) HE staining of neo-islet tissues between liver lobes on day 28. Neo-islet tissue (black arrowheads) and vascular networks containing blood cells (white arrowheads) are shown. (**C**) Double immune histochemical staining for insulin (green) and glucagon (red) of neo-islet tissues in liver on day 28. (**D**) Double immunohistochemical staining for insulin (green) and CD31 (red) on day 28. (**E**,**F**) Double immunohistochemical staining for insulin (green) and Ki-67 (red) on day 28. Arrows denote Ki-67/insulin double-positive β-cells in the neo-islet tissue. Bar: 50 mm. Adapted with permission [132]; published by The Japanese Society for Regenerative Medicine, 2018.

**Figure 6:**
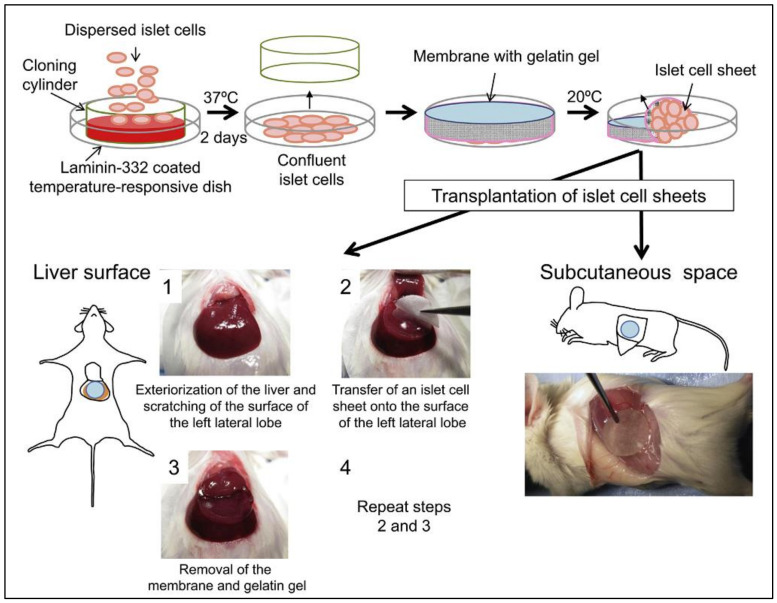
Schematic of the recovery and transplantation of rat islet cell sheets from a temperature-responsive dish (TRD) using a gelatin/membrane. Dispersed rat islet cells were seeded into a cloning cylinder, placed on a laminin-332-coated TRD and cultured for 2 days at 37 °C. The islet cell sheets were recovered from TRDs using gelatin/membranes and transplanted onto the liver surface or the subcutaneous spaces of STZ-induced diabetic SCID mice. Adapted with permission [132]; published by The Japanese Society for Regenerative Medicine, 2018.

The immunohistochemical study showed that the vascularized neo-islet tissues were produced on the liver surface to protect the viability and functions of islet cells (Figure 5). In contrast, the viable cells were not present in the subcutaneous space group [131]. In this subcutaneous space, the inherent confines include the absence of primary vascularization, local inflammation induction, and physical stress on the graft adequately hindered the engraftment and survival of transplanted islet cell sheets fabricated by 1.6 × 10^6^ islet cells (Figure 6) [132].

Although different sites have been analyzed as potent implantation destinations for islet grafts, the great extent of graft application and delivery related to infusion in the liver portal circulation has prompted this transplantation site of interest in clinical guidelines [133]. The two acknowledged methodologies involved implanting the unadulterated islets in the liver via the hepatic portal vein path, whereas surgical cannulation and laparotomy of the portal vein were frequently utilized as the primary islet transplant methods.

Recent conventions employed the percutaneous transhepatic (PT) strategy to graft donor islets in the cadaveric islet transplantation [134,135]. In contrast to the surgical laparotomy, this technique is insignificantly obtrusive and therefore is accomplished by local anesthesia added with hypnotics, and opiate analgesia recommended as premedication. Entry in the portal vein is accomplished by the PT approach utilizing the combination of fluoroscopy and ultrasound techniques. Briefly, a right portal vein part is cannulated, and a catheter is situated adjacent to the portal vein convergence, established with a portal venogram. The risk of portal vein blockage is diminished by adding heparin (70 U/kg) to the islet formulation. Then, the islets are aseptically infused to the chief portal hepatic vein under gravity, with regular checking of hepatic portal venous pressure (through an indirect pressure transmitter) previously, during, and after implantation. The medical ultrasound study should be conducted on the first day and after one week, then transplanted to eliminate idiopathic spontaneous intraperitoneal hemorrhage (ISIH) and ensure the general flow in the portal vein of the patient [136].

In an imminent clinical investigation, 20 young T1DM patients were enlisted and randomized to mesenchymal stem cell (MSC) management. One year follow-up exhibited residual β-cell activity estimated by serum C-peptide using a mixed-meal tolerance test (MMTT). No symptoms of MSC therapy were observed, which shows that this technique is a promising and safe methodology to intercede in disease growth and safeguard the function of β-cells [137]. Furthermore, an investigation detailed single-centered participation with suppressed T1DM development by combining autologous hematopoietic stem cell transplantation (aHSCT) and immunoablation in newly diagnosed individuals. During the average 52 weeks of development, 87% of patients remained deprived of the exogenous insulin for a minimum of 288 days. The concentration of average HbA1C was 10.9% initially and 5.9%, 6.4%, 6.8%, and 7.1% at 1, 2, 3, and 4 years, respectively, after aHSCT. Any extreme DM complexity was not observed. Although, it can prompt T1DM reduction with great BGL control in most of the patients, with the time of emission lasting more than 5 years in a few patients [138]. The basic mechanism of preventing endogenous β-cells mass utilizing MSC is not entirely elucidated presently [134].

Luu et al. recognized the benefit/risk recommendations of patients pre-transplant. Islet transplantation could prevent T1DM-based complications. The lack of BGL control caused due to hypoglycemia unawareness (HU), and diabetes was the main theme of pre-transplant. The authors identified four types of sub-themes: hypoglycemia terror, hopes after transplant, diabetes-related difficulties, and transplant results. Patients were terrified of long-lasting problems of pre-transplant and the expected cell islet transplant could be effective in diabetes treatment [139].

### 3.4. Artificial Pancreas

An artificial pancreas (AP) with a closed-loop structure has been an effective strategy to deal with diabetes [108]. The AP, an integrated system, monitors BGL automatically and yields insulin/insulin combinations to patients with T1DM. A potential AP acts as life-changing and novel for many T1DM patients [140]. The FDA accepted the first hybrid closed-loop system (Medtronic’s MiniMed 670G System) on 28 September 2016. It monitors BGL and manages basal insulin doses automatically in people with T1DM. The FDA supported and fostered this medical device as safe and efficient [141].

Oukes et al. developed a survey to study the associated technology’s readiness and community influence by accepting AP in the large sample as compared with invited and self-selected patients with T1DM. Their study demonstrated validity and reliability. This survey was accomplished by 109 invited and 425 self-selected persons. The intention of AP use was more remarkable in both groups; however, it was significantly greater in the self-selected respondents. The invited respondents showed discretion related to the compatibility of the product, the subsequent product complexity, the readiness of technology, and the usefulness of the product; the AP showed the advantage of technological innovation in self-selected respondents [142].

### 3.5. Tissue Engineering

In the National Science Foundation Assembly, tissue engineering (TE) was defined as the principle applications and approaches of life sciences and engineering for the fundamental knowledge of structure and function goals in pathological, normal mammalian tissues and the biologic substitutes development, which restore, improve or maintain the tissue function [143]. Normally, the endocrine pancreas comprises the islets of Langerhans, which are scattered in the exocrine pancreas. The islets are microorgans consisting of about 200 to 2000 endocrine cells, including the β-cells, alpha cells, delta cells, pancreatic polypeptide cells and epsilon cells. These cells are responsible for the secretion of insulin, glucagon, somatostatin, pancreatic polypeptide, and ghrelin, respectively [144].

Tissue engineering highlights another strategy for diabetes treatment. This field of research has numerous triumphs regarding the improvement of substitutes for the renovation of damaged tissues and also function restoration [145,146,147,148,149,150].

TE strategy is employed to fix damaged tissues by utilizing three components, for example cells (stem cells), signal molecules (growth factors) and scaffolds. These strategies are safe and comfortable for patients resulting in acceptable outcomes. TE-based therapy utilizing Plasma Rich Platelets (PRP), Peripheral Blood Mononuclear Stem Cells (PBMNCs) and Cord Blood Mononuclear Stem Cells (CBMNCs) effectively treated the unhealed diabetic injury [151].

Pancreatic Islet transplantation reinstates the β-cell accumulation in the T1DM or adjusts the decreased quantity of β-cells present in T2DM, enhancing the formation of insulin impedance [152]. Nonetheless, organ deficiencies and the prerequisite for immunosuppressive drugs for transplantation are the primary hurdles for an experimental study of this treatment [153,154]. The β-cells production from stem cells with a hematopoietic or embryonic source may be a perfect method to fabricate adequate amounts of β-cell mass. Alternatively, organ transplantation has become a feasible option for insulin treatment, which depends on transporting sufficient β-cell mass to reestablish normoglycemia. The whole organ pancreatic transplantation and islet transplantation are two parallel preventive methodologies employed to treat T1DM. The results of effective whole-organ pancreatic transplantation revealed that synchronous kidney and pancreas transplantation might improve the outcomes [153,154,155,156]. Furthermore, the authors demonstrated that the possibility of the draft survival during the principal time of the simultaneous transplantation of the pancreas and kidney, the pancreas after kidney transplantation, and the pancreas alone, was 89%, 86%, and 82% individually, but decreased to 71%, 65%, and 58% individually, 5 years after the transplantation. In this manner, numerous organ recipients were re-transplanted in a couple of years [157].

One of the promising methods used to provide insulin to T1DM patients is the transplantation of isolated pancreatic islets. Although this strategy showed short-term results in providing insulin independence to patients, its long-term success was limited. One main limitation of such an approach is the limited supply of donor cells, which requires xenotransplantation, ultimately resulting in the destruction of the transplanted islet cells by the recipient’s immune system, therefore necessitating a lifelong immunosuppressant drug therapy [158].

Xenotransplantation is acquainted as the key to deal with organ deficiency. However, there is an associated danger of viral transmission and graft rejection. Mainly, solid organ transplantation is related to different problems. For instance, transplant individuals can be affected with diabetes [159,160]. A large exposure to bacteria, viruses, and parasite infections is another factor of anxiety in this transplantation [161,162,163]. A similar process can influence islet transplantation which can obscure the engraftments. Some controller mechanisms and agents are associated with β-cell development (in vivo and in vitro). The transplanted islet cells developed on a 3D platform act as a boundary in contrast to the immune system and provide adequate transportation of supplements, oxygen, and waste materials [164]. Furthermore, TE was investigated to characterize the associated drugs and their utilization in the 3D microenvironment, which improved islet cell functionality and viability [165,166,167].

A TE methodology of culturing insulin-creating cells with the extracellular matrix (ECM) molecules in 3D constructs can possibly upgrade the adequacy of cell-based treatments for diabetes [168].

Some examinations noted that PEG hydrogel frameworks could mimic and provided cell–cell adherent intersections in the microenvironment of insulin-secreting β-cells [169,170,171]. Bernard et al. structured a culture cell framework using a PEG hydrogel microwell. The formed β-cells aggregated in the hydrogels indicated increased cell functionality and viability (insulin secretion) in contrast to single-cell cultures [169]. Kelly et al. formulated a PEG-extracellular matrix to mimic the interaction of the islet microenvironment. Therefore, the hydrogel comprising collagen type I and type IV, PEG, fibrinogen, fibronectin, vitronectin, collagen type IV, and laminin was prepared. The insulin secreting cells were encapsulated in the PEG-ECM hydrogel, and critical increments in cell feasibility and the extent of insulin emission were observed. The fundamental problem related to islet cell aggregation was hypoxia at the site because of a deficient oxygen level, prompting cell death and necrosis [167].

Pancreatic islet cells relocated inside a tissue-engineered framework could effectively engraft and accomplish insulin freedom in T1DM. In this regard, the tissue-engineered ‘mini pancreas’ accomplished long-term insulin freedom in a T1DM patient [172,173].

### 3.6. Gene Therapy

The intestinal cells, for example, enteroendocrine K-cells, indicate numerous resemblances with pancreatic β-cells and secrete glucose-dependent insulinotropic polypeptide, as well as comprising the necessary prohormone convertases for the processing of proinsulin to insulin [174]. Accordingly, many analysts endeavored to control K-cells (intestinal cells) in vitro and caue the secretion of insulin; nonetheless, the grafting of these cells was neglected to invert DM securely. The transgenic mice, altered to secrete insulin under GIP promoter, subsequently utilizing STZ to instigate diabetes, indicated an ordinary glucose level. This showed that K-cells delivered insulin in adequate quantities to maintain BGL [175].

The gene therapy method has been presented for the treatment of diabetes depending on the gene co-expression network (GCN) of both glucokinase and insulin qualities in skeletal muscles by adeno-associated viral vectors. Normal BGL can be accomplished by the longstanding viability of gene therapy except for the exogenous insulin supply [176]. These vectors cause a mild resistant reaction and contaminate both lethargic and partitioning cells without incorporating the host cell genome. Therefore, an adeno-associated viral vector is the best candidate for gene therapy. The adeno-associated viral vectors encode the glucokinase and insulin genes in the skeletal muscles of STZ-induced diabetic dogs and mice. Their co-expression upgrades the chromosomal translocation of glucokinase and GLUT4, which encourages glucose absorption in muscle cells. The expression of glucokinase suppresses the glucose phosphorylated to glucose-6-phosphate in skeletal muscle. In addition, the glucose sensor detects BGL and shows the quantity of insulin formed to accomplish normal BGL [177,178].

Li et al. utilized AAV serotype 2 as a medium, transfected with the duodenal and pancreatic homeobox 1 (pdx-1) gene, a crucial transcription factor in pancreatic islet development and differentiation. The presence of green fluorescent protein (GFP) expression affirmed that the quality of pdx-1 in the liver produced insulin ectopically for normoglycemia. The hyperglycemia in STZ-induced diabetic mice was decreased via AAV-pdx-1 gene therapy [179].

Neurogenin (Ngn3) is another gene necessary for endocrine pancreas formation. The pdx-1 promoter controls the forced expression of Ngn3, which is sufficient to encourage the production of all endocrine cells [180]. The hepatic cell transfection by adenovirus with Ngn3 prompts insulin formation and the trans-separation of the oval cell population [180]. The introduction of neuroD1 into the liver of STZ-induced diabetic mice leads to an increased response to a stimulus upstream and downstream of the pancreatic translating agents, involving Pax6, Nkx6.1, Ngn3, Nkx2.2, and Pax4 without significant hepatotoxicity [181,182]. In the main pancreatic duct cells, the NeuroD1 gene shows the most grounded impact in prompting insulin articulation in contrast with pdx-1, Ngn3, and Pax4 [183]. The developed DNA targets promoters in different cells, such as the cell-type choice, insulin-like growth factor binding protein-1 (IGFBP-1), liver-type pyruvate kinase (L-PK), phosphoenolpyruvate carboxykinase (PEPCK), glucose 6-phosphatase (G6Pase), egg whites, and S-14. The hepatic insulin gene therapy indicates insulin secretion; however, this secretion is often low due to the gene therapy’s elevating action in contrast to solid unregulated promoters, such as cytomegalovirus [184]. For instance, L-PK is employed to advance insulin articulation in the liver leading to glucose-responsive insulin and the reestablishing of normal BGL for as long as 1 month. The main restriction of L-PK is the limitation of this promoter by insulin. The egg whites and S14 may be utilized to maintain a strategic distance from this input [184].

The plasmid DNA injected into the muscles and liver of STZ-induced diabetic mice indicated normal glycemia for multi and 30 weeks, respectively. The co-infusion of plasmid DNA with insulin-associated furin resulted in significant dynamic insulin inside the muscle [185]. Bringing human insulin genes to the liver or pancreatic cells via ex vivo transfection and then autologous grafting demonstrated beneficial outcomes in pigs, as hyperglycemia, insulin emission, and diabetic complexities were strikingly enhanced for over 47 weeks [186]. However, this development does not proceed; gene silencing occurs because this method of gene silencing is not well-known. Another fruitful method is the administration of a lentivirus vector, which conveys an altered human insulin gene to the portal system of the liver of diabetic mice. As a result, this strategy enables liver cells to detect glucose, and thus synthesize, release, and store human insulin as a reaction [187].

### 3.7. Stem Cells Therapy

**Figure 7:**
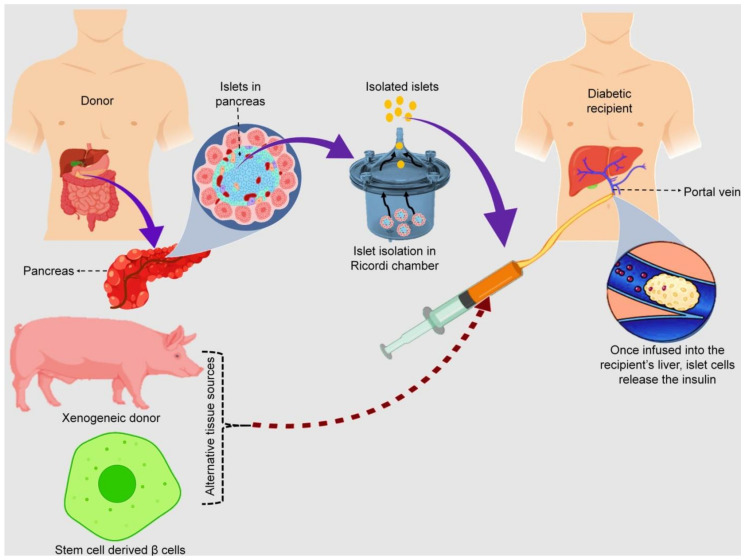
Transplantation and alternative sources of pancreatic islet cells.

Restrictions of the trans-separation of pancreatic progenitor cells are induced to investigate alternative sources of β-cells, for instance, embryonic stem cells (ESC) (Figure 7). All through embryonic improvement, the pancreatic epithelium is comprised of a multipotent progenitor developed into other types of pancreatic cell, for example, ductal, exocrine, and endocrine heredities [188]. The primary issue of this option is employing human stem cells amid the embryonic phase, which raises a moral issue, which undoubtedly becomes a constraint. Actuated pluripotent stem cells (iPSCs) are formed by reprogramming the somatic cell nucleus [189]. The iPSCs consist of comparative properties for ESCs and are formed by ribonucleic acids and small molecules. These SCs coordinate genetically with most of the patients while preventing immune reactions [190]. The iPSCs acquired from human pancreatic cells are formed by utilizing few transcription factors, such as lenti Sox2, Klf4, Oct3/4, and c-Myc, named human-induced pluripotent stem cells (hiPSCs). The hiPSCs indicated equivalent levels of explicit ESCs markers, quickly separated into three germ layers where the hiPSCs shape an embryoid structure [191].

Recent investigations announced conventions that fundamentally produced β-cells which were expressed to β-cells disconnected from humans [192]. After the transplantation in diabetic mice (post 14 days), the new microorganism determined that β-cells emitted insulin in light of higher glucose levels and maintained the normal BGL. This result could be improved by applying iPSCs or ESC as β-cell replacement treatments for diabetes patients in the future [193]. In any case, the iPSCs and ESCs differentiation force, numerous stages, and diverse variables should be considered. Moreover, the clinical preliminaries of the differentiated conventions were tedious, while the undifferentiated iPSCs and ESCs conveyed tumorigenesis hazards [194]. Curiously, pancreatic progenitor cells (PPCs) could somehow thrash the impediments faced by iPSCs and ESCs because of the closeness of pancreatic heredity [195].

Many investigations reference the advantages of mesenchymal stem cells (MSCs) in diabetes treatment by immunomodulatory methods. In this microenvironment, β-cells are recovered, and ruinous T-Helper1 cells are suppressed [196]. In these investigations, specialists found an enhancement in the control of hyperglycemia. Therefore, MSCs treatment can be used with other therapies. Practical histologic proof corroborates that undifferentiated MSCs do not recover new β-cells straightforwardly [197]. In addition, the media acquired from MSC cultures created powerful restorative outcomes upon injection into diabetic mice [198]. This methodology provides an effective treatment on account of its cell-less nature; as an outcome, it retains the issues of oncogenesis and autoimmunity. In the end, if the gathering systems are improved, MSC media can be utilized together with other standard and well-established treatments to reduce insulin reliance or the near transplantation of insulin-delivering cells obtained from other pluripotent sources [199].

Bone marrow undifferentiated cells (BMSC) are used to supplant harmed β-cells. However, they develop lower amounts of insulin. Luckily, fat tissue has less immunogenicity, more immunomodulatory, and can create more foundational microorganisms than bone marrow [200]. Moreover, fat determined foundational microorganisms (ADSCs) portrayed by their straightforward segregation system, easy availability, and the limit of the undifferentiated organisms’ multiplication are not influenced by the patient’s age [201]. In an examination, ADSCs separated into insulin-pancreatic cells (IPCs). At this point, IPCs were infused in the pancreas of STZ-induced mice. The histopathological examination uncovered the enlistment of the pancreatic recovery process with various expanded clogged blood vessels and diffused multiplied islet cells. The separated IPCs transplantation enhanced the morphology and capacity of pancreatic islet cells in diabetes-induced mice. Further investigations were completed to affirm the ability of ADSCs in enhancing T2DM. The ADSCs implantation could reestablish islet β-cells and improve hyperglycemia through islet angiogenesis upgrade, lessen cell apoptosis, and improve insulin affectability [202]. Another study uncovered that utilizing human eyelid fat, tissue-inferred undeveloped cells offered expanded human insulin and C-peptide levels in T2DM mice and diminished IL-6 and triglyceride in the T2DM mice model [203]. The first preliminary actuating BM-MSC, which separated into IPCs, was finished using adenoviral vectors coding for mouse pdx-1. Formed IPCs demonstrated an ability to free insulin in a glucose-subordinate way [204,205].

Various examinations were completed on the umbilical rope (UC)-MSCs, particularly Wharton’s jam-inferred MSCs (WJ-MSCs) and BM-MSCs. The WJ-MSCs demonstrated a predominance in separation potential for developed β-cell phenotypes than in BM-MSCs [206]. Furthermore, WJMSCs uncovered a more noteworthy limit of mRNA articulation of insulin and C-peptide than BMMSCs [207]. Thus, ADSCs can be a hopeful restorative method for patients with T1DM and T2DM if their safety and adequacy are affirmed [166].

Human placenta-derived MSC (PD-MSC) also enticed researchers because of their capacity to create insulin. Therefore, the pilot contemplates attempted the impact of PD-MSC after intravenous infusion for 10 T2DM patients. The result showed a huge abatement of glycosylated hemoglobin in all ten patients, and the dimensions of insulin and C-peptide were greater in contrast to earlier medicines. Moreover, no advancement of reactions, for example, chills, liver harm, fever, and insusceptible dismissal were observed, and the heart and renal capacities improved [208].

Undifferentiated cells are specific cells inside tissues, having two specific characteristics of self-reestablishment and capacity to be divided into a wide assortment of cell types with specific heredities [208]. Their ability to separate into an ancestor cell that does not divide further while producing another cell that divides or develops into specific cells, provides their stemness. Undifferentiated organisms show diverse properties relying upon their time and the source of gathering, as well as their abilities to produce distinctive ancestries (pluripotent versus multipotent). Extensively, these cells are named embryonic foundational microorganisms and grown-up undifferentiated organisms. In a suitable culture condition, undeveloped embryonic cells are equipped for duplicating inconclusively and are pluripotent, i.e., offering to ascend to specific cells. Grown-up undifferentiated cells or substantial immature microorganisms are commonly multipotent and equipped for producing an ancestries subset, generally limited to the tissue from which these cells are derived. The capacity of undifferentiated organisms to separate into various genealogies provide these cells’ attractive application in numerous treatments, for example: tissue recovery, neurodegeneration, cardiovascular infections, osteoarthritis, rheumatoid joint inflammation, and diabetes [209].

## 4. Conclusions and Future Prospects

Diabetes treatment is in desperate need of change. Diabetes occurrence is rising at an unprecedented rate, and diabetes poses immense economic pressure in terms of the potential spending on healthcare and the cost of treating diabetic complications. While there are many relevant new therapeutic agents, none are effective alone (except insulin or insulin analogs) to achieve adequate glycemic control. Therefore, combinations of complementary drugs perform an increasingly important function in disease treatment. In addition, the emergence of new drugs targeting additional targets will improve our ability to treat this critical disease successfully.

## Figures and Tables

**Table 1:** Nanocarriers-based therapy of diabetes and associated complications.

Nanocarriers	Composition	Disease	Advantageous Features/Findings	Reference
Polymeric NPs	Metformin (MET)-loaded polymeric NPs	Diabetes mellitus	Controlled drug release profile as compared to pure MET Lowering the blood glucose levels	[36]
Phyto-NPs	Selenium cleome droserifolia NPs	Diabetes mellitus	Significant elevation in serum glucose concentration Decrease in serum insulin	[37]
Zinc oxide NPs (ZnO NPs)	*Costus igneus*-loaded ZnO NPs	Diabetes mellitus	High antidiabetic activity of *Costus igneus*-ZnONPs	[38]
Dendrimer	G4 PAMAM	Diabetes mellitus	Decreased glycoxidation Post-synthetic non-enzymatic alteration of biomacromolecules in sustained streptozotocin diabetic rodents	[39]
Solid lipid nanoparticles (SLN)	Valsartan (Val)-loaded SLN (Val-SLN)	Diabetic foot ulcer	Small particle size High entrapment efficiency Sustained drug release	[40]
Bovine serum albumin NPs (BSA-NPs)	Apatinib-loaded BSA-NPs coated hyaluronic acid (HA) (Apa-HA-BSA-NPs)	Diabetic retinopathy (DR)	Sustained drug release No cytotoxic effect on rabbit corneal epithelial Cells (RCE) Improved retinal accumulation	[41]
Gold NPs (AuNPs)	Streptozotocin (STZ) -loaded AuNPs	Diabetic cardiomyopathy	Decreased myocardial mRNA and protein tissue content of transforming growth factor β1 (TGF-β1) Potential therapeutic effect in the treatment of early DCM	[42]
Chitosan NPs (CSNPs)	Polydatin-loaded CSNPs (PD-CSNPs)	Diabetic cardiomyopathy	Significant reduction in glucose and glycosylated haemoglobin levels Improvements in heart function biomarkers were achieved by lowering serum creatine kinase and creatine kinase myocardial band activity	[43]
Liposome	Fibroblast growth factor 1 (FGF1) loaded liposome	Diabetic cardiomyopathy	Improved myocardial function Improved apoptosis of cardiac cells Increased myocardial blood flow	[44]
Liposome	Calycosin-loaded nanoliposomes	Diabetic nephropathy	Regulated the function of mitochondria in kidney cells, ROS production, viability and lipid peroxidation of diabetic rats	[45]
Poly(lactic-co-glycolic acid) (PLGA) NPs	Crocetin-loaded PLGA-NPs	Diabetic nephropathy	Antifibrosis and anti -inflammatory effects	[46]
Bilosomes	Eprosartan mesylate loaded-bilosomes	Diabetic nephropathy	Lowering blood pressure Reducing the degree of fibrosis	[47]
Silver NPs (AgNPs)	*Allium cepa* extract-loaded AgNPs	Diabetic neuropathy	Higher level of α-amylase and α-glucosidase inhibitory activities Better antioxidant activity and less cytotoxicity	[48]
Transferosomes	Pioglitazone and eprosartan mesylate-loaded nano-transferosomes	Coexisted hypertension with Type 2 diabetes	Prolonged management of diabetes and hypertension as compared with oral drug	[49]
Exosome	RNA interference (RNAi)-based exosome	Diabetic chronic wound	Engineered miR-31 exosomes promoted diabetic wound healing by enhancing angiogenesis, fibrogenesis and reepithelization	[50]

## Data Availability

Not applicable.
